# Diffusion-weighted MRI for predicting treatment response in patients with nasopharyngeal carcinoma: a systematic review and meta-analysis

**DOI:** 10.1038/s41598-021-98508-5

**Published:** 2021-09-23

**Authors:** Min Kyoung Lee, Yangsean Choi, So-Lyung Jung

**Affiliations:** 1 Department of Radiology, Yeouido St. Mary’s Hospital, College of Medicine, The Catholic University of Korea, Seoul, Republic of Korea; 2 Department of Radiology, Seoul St. Mary’s Hospital, College of Medicine, The Catholic University of Korea, Seoul, Republic of Korea

**Keywords:** Oncology, Cancer, Cancer imaging, Head and neck cancer

## Abstract

Early prediction of treatment response in nasopharyngeal carcinoma is clinically relevant for optimizing treatment strategies. This meta-analysis was performed to evaluate whether apparent diffusion coefficient (ADC) from diffusion-weighted imaging (DWI) can predict treatment response of patients with nasopharyngeal carcinoma. A systematic search of PubMed-MEDLINE and Embase was performed to identify relevant original articles until July 22, 2021. We included studies which performed DWI for predicting locoregional treatment response in nasopharyngeal carcinoma treated with neoadjuvant chemotherapy, definitive chemoradiation, or radiation therapy. Hazard ratios were meta-analytically pooled using a random-effects model for the pooled estimates of overall survival, local relapse-free survival, distant metastasis-free survival and their 95% CIs. ADC showed a pooled sensitivity of 87% (95% CI 72–94%) and specificity of 70% (95% CI 56–80%) for predicting treatment response. Significant between-study heterogeneity was observed for both pooled sensitivity (*I*^2^ = 68.5%) and specificity (*I*^2^ = 92.2%) (*P* < 0.01). The pooled hazard ratios of low pretreatment ADC for assessing overall survival, local relapse-free survival, and distant metastasis-free survival were 1.42 (95% CI 1.09–1.85), 2.31 (95% CI 1.42–3.74), and 1.35 (95% CI 1.05–1.74), respectively. In patients with nasopharyngeal carcinoma, pretreatment ADC demonstrated good predictive performance for treatment response.

## Introduction

Nasopharyngeal cancer (NPC) is a common subtype of head-and-neck cancers prevalent in Asia and Africa, with about 129,000 new cases reported in 2018^1^. NPC is widely treated using intensity-modulated radiotherapy and/or chemotherapy. Although early-stage NPC is adequately treated using radiotherapy alone, chemotherapy is required for locoregionally advanced disease^1^. However, patients with locoregionally advanced NPC have a high probability of recurrence with poor prognosis, even after chemoradiotherapy^2^. Thus, early prediction of treatment response and identification of patients with treatment resistance are clinically relevant for individualized treatment planning.

The International Union Against Cancer/American Joint Committee on Cancer TNM staging system is used to classify NPC^3^. Because of the superior soft-tissue resolution and diagnostic performance in the T stage compared to other modalities, MRI is the imaging modality of choice for primary tumor evaluation^4^. Furthermore, MRI is valuable for tumor staging and post-treatment tumor response evaluation.

However, conventional MRI has limitations regarding the early prediction of treatment response in NPC^5^. Advanced imaging techniques, including DWI and perfusion imaging, are usually applied for the evaluation of treatment response and residual or recurred tumor^6^. Importantly, DWI is a common imaging technique for assessing tissue microstructure by measuring tissue water diffusivity^7^. The ADC, a quantitative parameter calculated from DWI, reflects tumor microstructure, and pretreatment ADC has shown promise in tumor staging and predicting treatment response^8–10^. The benefits of DWI for predicting treatment response and prognosis in NPC have been reported^5,6,9,11–19^; however, the reported sensitivities and specificities were variable. Thus, the current study sought to bridge the gap in the available literature.

The purpose of this systematic review and meta-analysis was to assess the predictive performance of pretreatment ADC for treatment response in patients with NPC.

## Materials and methods

This systematic review and meta-analysis was conducted according to the Preferred Reporting Items for Systematic Reviews and Meta-Analyses (PRISMA) guidelines^20^. The institutional review board of our institution approved this study.

### Literature search strategy

A search of the PubMed-MEDLINE and EMBASE databases was performed to identify relevant original articles on the use of DWI MRI for predicting locoregional treatment response in NPC treated with neoadjuvant chemotherapy, definitive chemoradiation therapy, or radiation therapy up until July 22, 2021. The following search terms were used: [(nasopharyngeal)] AND [(carcinoma) OR (carcinomas) OR (cancer) OR (cancers) OR (squamous cell carcinoma)] AND [(chemoradiation) OR (chemoradiotherapy) OR (radiotherapy) OR (radiation therapy)] AND [(“diffusion weighted”) OR (“diffusion-weighted”) OR (dw-mri) OR (DWI) OR (“apparent diffusion coefficient”) OR (ADC) OR (“intravoxel incoherent motion”) OR (IVIM)]. Only studies published in English were included. We defined ‘predictive’ as a biomarker of the treatment response to therapy and ‘prognostic’ as a biomarker of the final survival outcome^21^. The search was limited to studies involving human patients. The bibliographies of the selected articles were further screened to identify other potentially relevant articles.

### Inclusion and exclusion criteria

The inclusion criteria were: (1) population: patients with histologically proven NPC who underwent neoadjuvant chemotherapy, definitive chemoradiation, or radiation therapy; (2) index test: DWI MRI with provision for pretreatment ADC of primary NPC; (3) reference standard: the reference standards of the treatment outcome as determined by histology, clinical/imaging follow-up, or a combination of these; (4) outcomes: locoregional failure after neoadjuvant chemotherapy, definitive chemoradiation, or radiation therapy reported in sufficient detail; and (5) study design: all observational studies (retrospective or prospective).

The exclusion criteria were: (1) case reports, review articles, editorials, letters, and conference abstracts; (2) insufficient data on locoregional failure and control; (3) did not provide ADC values of primary NPC; (4) insufficient detail to produce 2 × 2 tables; and (5) overlapping patients and data. For population overlap, the study with the larger cohort was included. Two reviewers (blinded and blinded) independently selected appropriate study reports using a standardized form. Disagreement was resolved by reaching a consensus after discussion with a third reviewer (blinded).

### Data extraction

The following information was extracted into a standardized form: (1) study characteristics: first author, year of publication, affiliation, patient enrollment period, number of patients, and study design; (2) clinical information: age, cancer stages, endpoints, treatment, criteria for treatment response, and follow-up period; and (3) MRI acquisition parameters: manufacturer, model, tesla, time repetition, time echo, field of view, matrix, b-values, ADC threshold values, ADC change between treatment, and method of delineating region of interest.

### Quality assessment

Two reviewers (blinded and blinded) with seven years of experiences in the head and neck diagnostic radiology independently extracted the data and performed a quality assessment. The included studies were evaluated using the Quality Assessment of Diagnostic Accuracy Studies-2 (QUADAS-2) criteria^22^ for predictive studies and Quality in Prognosis Studies (QUIPS) for prognostic studies^23^. Data extraction and quality assessment were performed independently by two reviewers (blinded and blinded). Any disagreement was resolved by a consensus.

### Data synthesis and statistical analyses

Using random-effects modeling, pooled sensitivity and specificity with 95% CIs were generated from individual predictive studies; similarly, pooled overall survival (OS), local relapse-free survival (LRFS), and distant metastasis-free survival (DMFS) with 95% CIs were generated from prognostic studies. For predictive studies, hierarchical summary receiver operating characteristic (HSROC) curves with 95% CIs and predictive regions were graphically visualized. Publication bias was evaluated via Deeks’ funnel plot; Deeks’ asymmetry test was used to determine the statistical significance of publication bias^24^. Between-study heterogeneity was evaluated using Cochran’s Q test with statistical significance at *P* < 0.05^25^; the degree of heterogeneity was based on the Higgins inconsistency index (*I*^*2*^) where an *I*^*2*^ of 0–40%, 30–60%, 50–90%, and 75–100% indicate insignificant, moderate, substantial, and considerable heterogeneity, respectively^26^.

The threshold effect, a positive correlation between sensitivity and the false-positive rate, was visually assessed by the inspection of coupled forest plots of pooled sensitivity and specificity in which an inverted V-shape would indicate a threshold effect. Additionally, Spearman’s correlation coefficient of sensitivity and false-positive rates was calculated with a value of > 0.6 regarded to indicate a threshold effect^27^.

For predictive studies, subgroup bivariate meta-regression analyses were performed to determine the causes of heterogeneity across the studies according to the following covariates: (1) year of publication (after 2016 vs. before 2016), (2) study design (retrospective or prospective), (3) follow-up of patients (reported vs. not reported), (4) number of patients (> 60 vs. ≤ 60), (5) MR tesla (3.0-T vs. 1.5-T), 6) number of b-values used (≥ 4 vs. < 4), (7) radiologists blinded to outcome (blinded vs. not reported), (8) type of treatment received (concurrent chemoradiation therapy only vs. inclusion of induction or neoadjuvant chemotherapy), (9) proportion of patients with advanced T stage (T3/4) (> 70% vs. ≤ 70%), (10) proportion of patients with advanced N stage (N2/3) (> 70% vs. ≤ 70%), and (11) region of interest selection (single section vs. volume).

All statistical analyses were performed using STATA version 16.0 (StataCorp, College Station, TX, USA) and R version 3.6.2 (R Foundation for Statistical Computing, Vienna, Austria). *P* < 0.05 was considered statistically significant.

## Results

### Literature search

**Figure 1:**
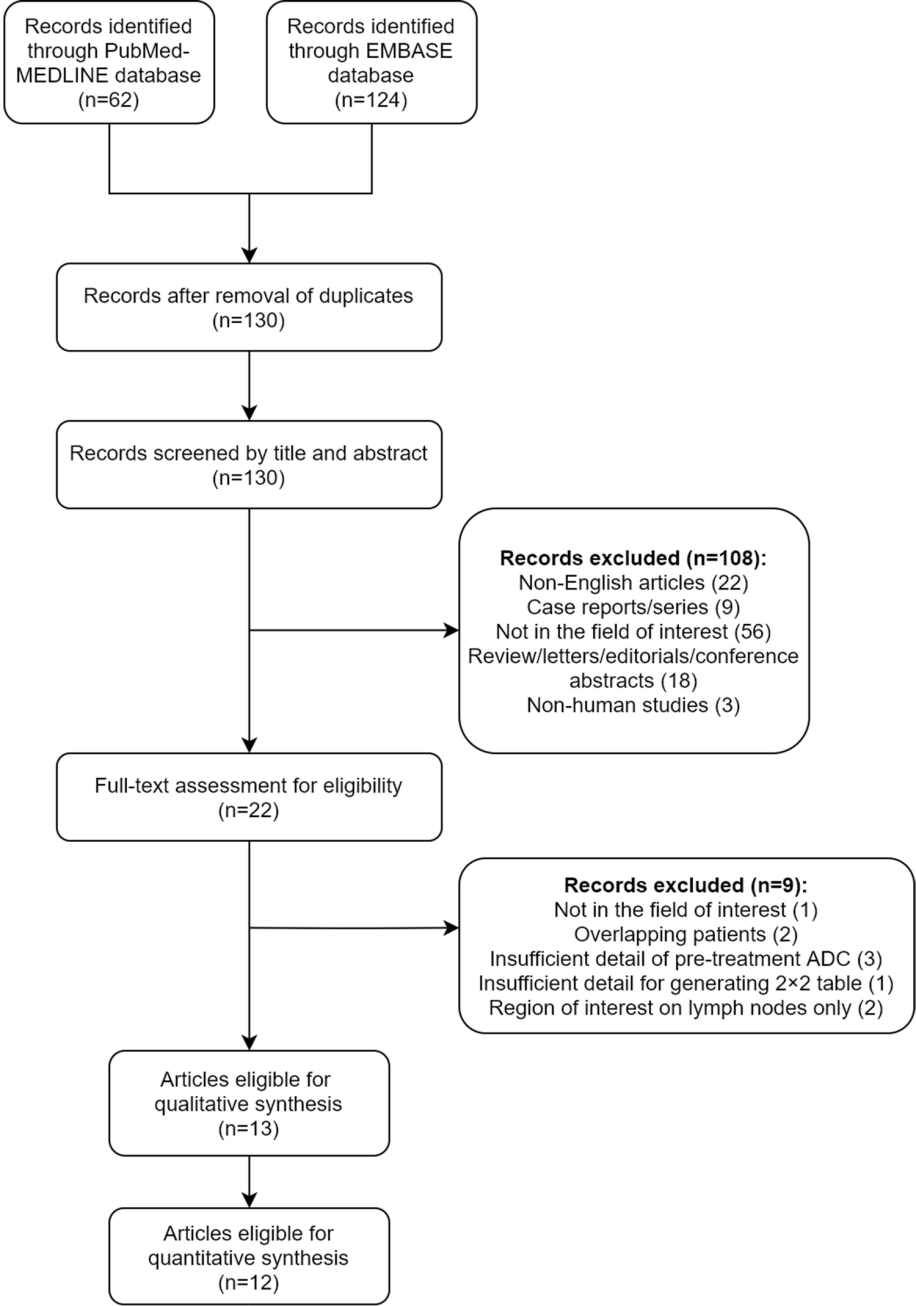
Flow diagram depicting the study eligibility criteria.

The initial search yielded 186 articles; 56 duplicates were removed. After screening the titles and abstracts, 22 articles were considered potentially eligible after excluding 108 articles for the following reasons: non-English articles (n = 22), case reports (n = 9), not in the field of interest (n = 56), non-original articles (i.e., reviews, letters, editorials, conference abstracts) (n = 18), and non-human studies (n = 3). After full-text review, nine articles were further excluded because they were not in the field of interest (n = 1)^28^, had overlapping study populations (n = 2)^10,29^, had insufficient information for the reconstruction of 2 × 2 tables (n = 1)^30^, insufficient detail of pretreatment ADC (n = 3)^31–33^, or region of interest was on lymph nodes only (n = 2)^34,35^. Thirteen original studies were included for qualitative synthesis^5,6,8,9,11–19^. Ultimately, 12 original articles were included for quantitative synthesis^5,6,9,11–19^. Studies with the primary aim of predicting treatment response^5,6,8,9,11,12,14,15,17,19^ and predicting prognosis of patients^13,16^ were evaluated separately in the meta-analysis; one study predicted both treatment response and prognosis^18^ (Fig. 1).

### Study characteristics

The total number of patients in all the studies was 2192 (715 for predictive studies^5,6,8,9,11,12,14,15,17,19^, 634 for prognostic studies^13,16^, and 843 patients for the predictive and prognostic study^18^ (Table 1). The number of patients in individual studies ranged from 36–843. The mean age of the patients ranged from 42.2–52 years. Nine studies were retrospective^8,9,11–13,16–19^, and four were prospective^5,6,14,15^. All studies originated from China except one from Israel^11^. T stages were reported in all studies while two studies did not report the N stage^8,19^; eight studies did not report M stages^8,11,13,14,16–19^. In four studies, concurrent chemoradiation was the only treatment modality^6,11,14,15^, whereas other studies used mixed treatment regimens in addition to radiation therapy, including induction chemotherapy^5,8,18^ and neoadjuvant chemotherapy^9,12,17^. The characteristics of the MRI examinations of the included studies are summarized in Table 2.

**Table 1:** The clinical characteristics of the included studies.

First author (year of publication)	Affiliation	Study design	Enrollment period	No. of participants	Mean age	Type of treatment received	Study aim	Criteria for treatment response	Advanced T-stage (T3/4)*	Advanced N-stage (N2/3)*	Distant metastasis (M1)*
Hirchoren (2019)	Jerusalem, Israel	Retrospective	January 2007-December 2017	58	47.7	CCRT	Response prediction	NA	48%	88%	NA
Tu (2019)	Zhongnan Hospital of Wuhan University, Wuhan University, China	Retrospective	December 2015-March 2017	36	48.5	NAC and/or IMRT	Response prediction	RECIST 1.1	67%	92%	8%
Qin (2018)	Central South University and Hunan Cancer Hospital, China	Prospective	December 2016-April 2017	81	48.4	IC or CCRT	Response prediction	RECIST 1.1	60%	90%	2%
Yan (2017)	The First Affiliated Hospital of College of Medicine, Zhejiang, China	Retrospective	NA	93	52	IMRT or CCRT	Survival prognosis	NA	32%	72%	NA
Hou (2016)	Hunan Cancer Hospital and the Affiliated Cancer Hospital of Xiangya School of Medicine, China	Prospective	April 2014-May 2015	43	48	CRT	Response prediction	NA	95%	72%	0%
Liu (2015)	Shandong Cancer Hospital and Institute, Jinan, China	Prospective	March 2014-November 2015	42	50	CRT	Response prediction	RECIST 1.1	64%	64%	NA
Xiao-ping (2015)	The third Xiangya Hospital, China	Prospective	April 2014-December 2014	50	48.9	CRT	Response prediction	RECIST 1.1	78%	74%	38%
Zhang (2015)	Sun Yat-sen University Cancer Center, China	Retrospective	November 2010-May 2012	541	45.3	RT or CRT	Survival prognosis	NA	66%	24%	NA
Chen (2015)	Fujian Provincial Cancer Hospital, China	Retrospective	September 2013-May 2014	59	45.2	NAC with IMRT	Response prediction	RECIST 1.1	78%	81%	NA
Zheng (2013)	Fujian Provincial Cancer Hospital, China	Retrospective	January 2007-June 2011	54	42.2	NAC and/or RT	Response prediction	WHO	74%	76%	39%
Huang (2019)	Sun Yat-sen University Cancer Center, China	Retrospective	April 2009-July 2014	843	44	CCRT and/or IC	Survival prognosis	NA	86%	44%	NA
Law (2016)	Prince of Wales Hospital, China	Retrospective	March 2004-April 2009	158	50	RT or CRT	Response prediction	Definition of local failure***	53%	NA	NA
Hong (2013)**	First Affiliated Hospital of Fujian Medical University	Retrospective	April 2010-November 2011	134	47 (median)	RT (13) or CRT (121)	Response prediction	WHO	55%	NA	NA

CCRT, concurrent chemoradiation therapy; NAC, neoadjuvant chemotherapy; IMRT, intensity-modulated radiation therapy IC, induction chemotherapy; CRT, conformal radiation therapy; RT, radiation therapy; NA, not applicable.

*Proportion of patients.

**Not included in quantitative data synthesis.

***Defined as histologically positive or increase in tumor size on imaging or endoscopic examination.

**Table 2:** MRI characteristics of the included studies.

First author (year of publication)	Manufacturer	MR tesla	TR/TE	Acquisition matrix	FOV, mm^2^	b-value	ROI selection criteria*	ROI exclusion criteria	ADC thresholds (× 10^-3^mm^2^/s)	ADC change between treatment (%)
Hirchoren (2019)	Siemens (Avanto), Siemens (Trio Tim), or Philips (Ingenia)	1.5 or 3.0	4000/78	96 × 96	490 × 230	0, 500, 1000	Single section	Cystic and necrotic regions	0.805	NA
Tu (2019)	Siemens (Prisma)	3.0	3000/62.4	44 × 90	280	0, 1000	Volume	Skull base and muscle	0.875	50% (AUC: 0.833)
Qin (2018)	GE (Optima)	1.5	4225/106	128 × 130	200	0,50,80,100,150,200,400,600,800,1000	Volume	NA	0.902	NA
Yan (2017)	Philips (Intera)	3.0	2947.1/43.3	96 × 96	220	0, 1500	Volume	Cystic and necrotic regions	0.72	NA
Hou(2016)	GE (Optima)	1.5	4225/106	128 × 130	260 × 260	0,50,80,100,150,200,400,600,800,1000	Volume	Necrotic regions and adjacent structures	0.898	NA
Liu (2015)	Philips (Achieva)	3.0	10,201.5/45	NA	220	0, 800	Single section	Cystic and necrotic regions	NA	NA
Xiao-ping (2015)	GE (Optima)	1.5	4225/106	128 × 130	NA	0,50,80,100,150,200,400,600,800,1000	Volume	Necrotic regions and adjacent structures	0.879	NA
Zhang (2015)	Siemens (Trio Tim)	3.0	5100/96	192 × 192	240	0, 1000	Volume	Cystic and necrotic regions	0.747	NA
Chen (2015)	Philips (Achieva)	3.0	4190/69	224	230 × 240	0,500,1000,1500	Single section	Necrotic regions	0.679	51% (AUC: 0.704)
Zheng (2013)	GE (Signa)	1.5	6000/the default minimum	64 × 64	240 × 240	0, 800	Volume	NA	0.916	NA
Huang (2019)	Siemens (Trio Tim)	3.0	5600/93	192 × 192	240 × 240	0, 1000	Single section	Cystic and necrotic regions	0.785	NA
Law (2016)	Philips (Intera)	1.5	2000/75	112 × 112	230	0,100,200,300,400,500	Volume	NA	0.55	NA
Hong (2013)**	GE (Signa)	1.5	6000/default minimum	128 × 128	24 × 24	0, 800	Single section	NA	NA	52.7% (AUC: 0.675)

NA, not applicable; ROI, region of interest; ADC, apparent diffusion coefficient; FOV, field of view; AUC, area under the curve for predicting treatment response.

*Single section = the ROI was drawn at the largest cross-sectional area of the tumor; Volume = the mean of all ADC values obtained from all sections involving tumor.

**Not included in quantitative data synthesis.

### Quality assessment

For QUADAS-2, all studies showed low risks of bias in flow and timing and patient selection domains; two studies had unclear risks of bias and unclear concerns regarding applicability in the domains of index test and reference standard^5,6^. For QUIPS, one study had a moderate risk of selection bias (Supplemental Figure 1)^13^.

### Predictive performance of DWI-MRI

**Figure 2:**
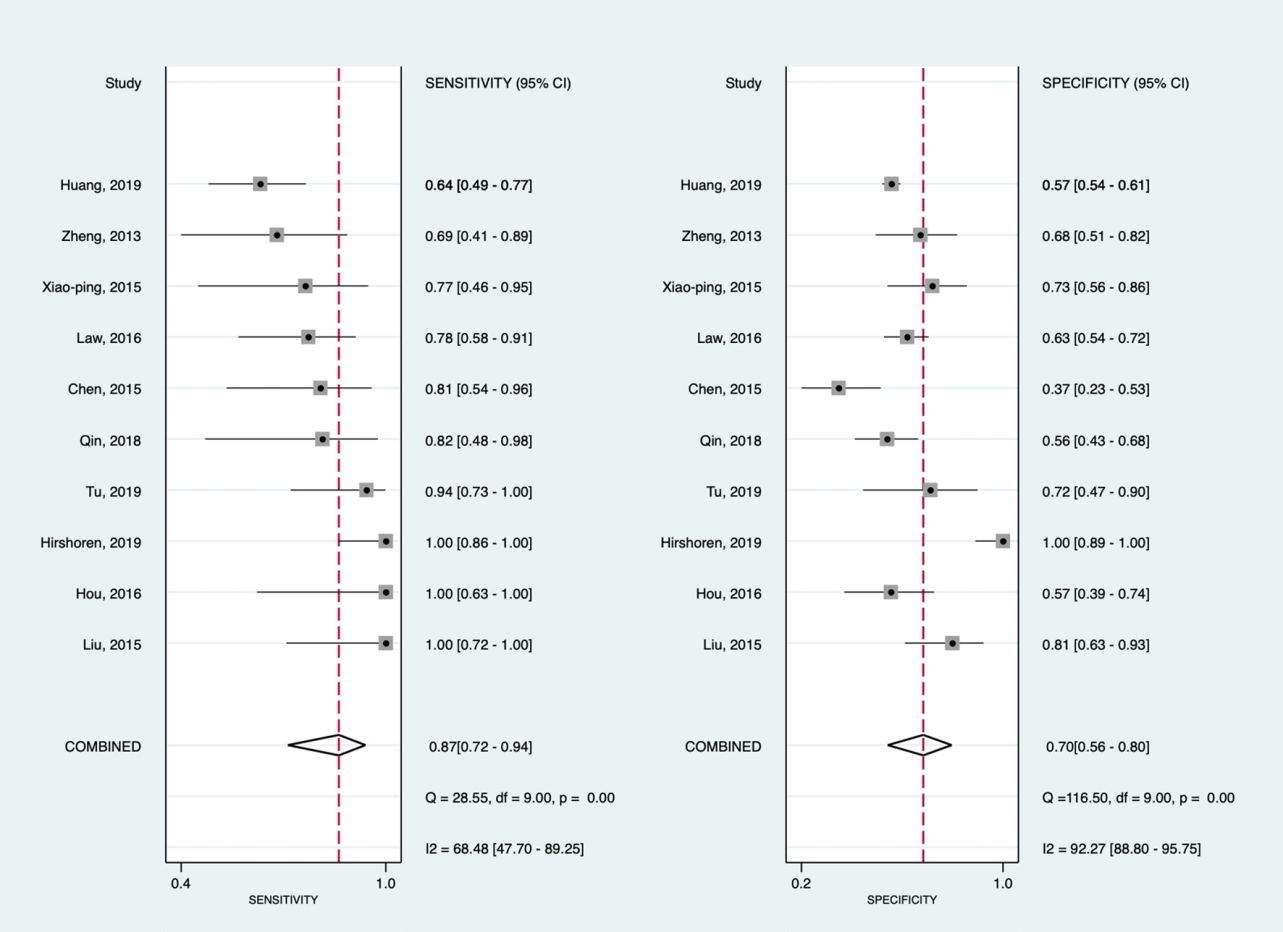
Coupled forest plots illustrating pooled sensitivity and specificity of pretreatment ADC for predicting treatment response in patients with nasopharyngeal carcinoma. Horizontal lines indicate 95% CIs of each study.

**Figure 3:**
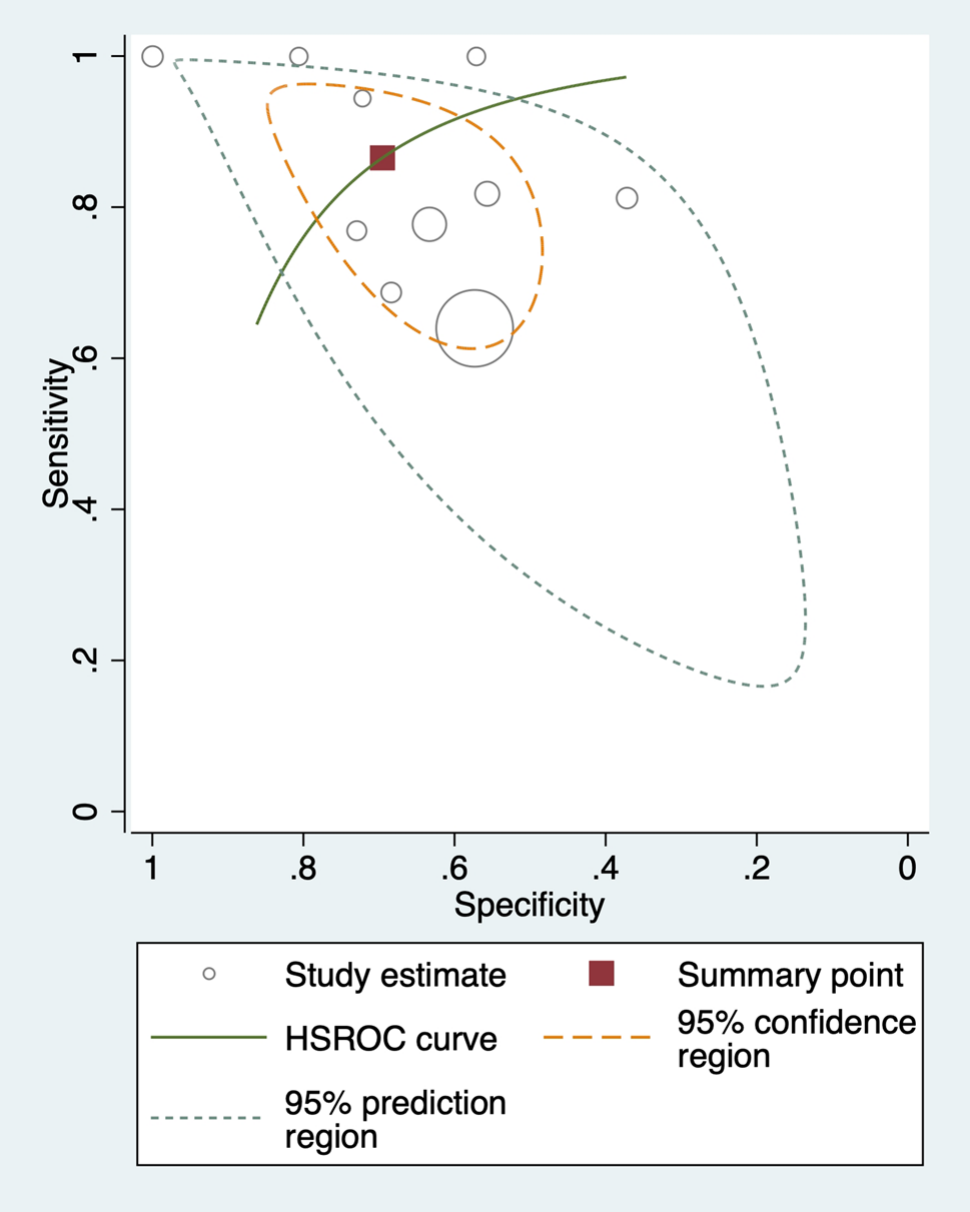
Hierarchical summary receiver operating characteristic (HSROC) curves of pretreatment ADC for predicting treatment response in patients with nasopharyngeal carcinoma.

Among predictive studies investigating treatment response predictions, DWI showed a pooled sensitivity of 87% (95% CI 75–94%) and specificity of 70% (95% CI 56–80%) (Fig. 2). Between-study heterogeneities were present according to the Q test (*P* < 0.01); particularly, the *I*^2^ statistic revealed substantial heterogeneity in the pooled sensitivity (*I*^2^ = 68.5%) and specificity (*I*^2^ = 92.3%). However, visual assessment of the coupled forest plot showed no threshold effect, and Spearman’s correlation coefficient of sensitivity and false-positive rates also indicated the lack of a threshold effect (-0.48 [95% CI -0.85–0.22]). In the HSROC curve, a large difference was observed between the areas of 95% confidence and prediction regions, suggesting between-study heterogeneities (Fig. 3). Based on the slope coefficient of Deeks’ funnel plot, the publication bias was low (*P* = 0.24) (Supplementary Figure 2). In three studies that reported the thresholds of ADC changes between treatment^8,12,17^, the sensitivities, specificities, and AUCs for predicting treatment response ranged 64–94%, 56.3–72%, and 0.675–0.833, respectively (Table 2).

### Subgroup bivariate meta-regression analyses of predictive studies

Table 3 shows the results of subgroup bivariate meta-regression analyses for determining the causes of between-study heterogeneity. Studies with no heterogeneity (*I*^2^ = 0%) had the following characteristics: (1) inclusion of > 60 patients; (2) at least four b-values for ADC mapping; (3) > 70% patients with advanced T stage (T3/4); (4) ≤ 70% patients with advanced N stage (N2/3); and (5) region of interest selection as either single section or volume. Prospective studies showed little heterogeneity (*I*^2^ = 3.7%).

**Table 3:** Subgroup meta-regression analyses for identifying heterogeneity.

Covariate	No. of studies	Sensitivity (95% CI)	Specificity (95% CI)	*I*^2^
**Year of publication**				
> 2016	4	86.1% (55.4–96.8%)	69.8% (51.6–83.3%)	48.5
≤ 2016	6	76.9% (66.7–84.7%)	62.9% (50.9–73.5%)	9.88
**MR tesla**				
1.5	4	83.0% (60.0–94.1%)	62.3% (43.0–78.3%)	27.92
3.0	6	77.3% (67.0–85.1%)	62.9% (57.4–68.1%)	42.28
**Number of patients**				
> 60	3	71.6% (57.7–82.3%)	58.9% (54.8–62.9%)	0
≤ 60	7	85.2% (71.8–92.8%)	68.9% (53.1–81.2%)	30.2
**Follow-up of patients**				
Reported	4	81.0% (51.5–94.5%)	72.8% (47.3–88.8%)	64.58
Not reported	6	83.8% (72.9–90.8%)	62.0% (48.7–73.7%)	4.07
**Number of b-values used**				
≥ 4	5	78.6% (67.8–86.4%)	57.4% (46.6–67.7%)	0
< 4	5	87.0% (65.1%-96.0%)	73.8% (58.7%-84.9%)	40.9
**Study design**				
Retrospective	6	78.7% (63.8–88.5%)	65.7% (49.2–79.1%)	52.93
Prospective	4	81.9% (66.3–91.2%)	65.4% (53.0–76.0%)	3.66
**Blinded to outcome**				
Yes	6	85.1% (73.7–92.1%)	64.4% (46.5–79.1%)	34.7
Not reported	4	74.3% (59.5–85.1%)	64.7% (55.6–72.8%)	28.2
**Treatment**				
CCRT or RT	5	86.6% (69.2–94.9%)	72.6% (58.3–83.4%)	33.3
IC or NAC included	5	74.1% (61.2–83.9%)	56.4% (46.6–65.9%)	12.9
**Proportion of advanced T-stage (T3/4)**				
> 70%	5	69.3% (59.2–77.8%)	57.8% (46.5–68.4%)	0
≤ 70%	5	89.6% (73.2–96.4%)	72.5% (57.3–83.8%)	36.8
Proportion of advanced N-stage (N2/3)*				
> 70%	7	80.2% (70.3–87.4%)	62.7% (50.1–73.7%)	35.8
≤ 70%	2	84.7% (30.3–98.6%)	68.4% (43.3–86.1%)	0
**ROI selection**				
Single section	3	93.6% (69.7–98.9%)	80.0% (26.8–97.8%)	0
Volume	7	74.3% (64.7–82.1%)	61.1% (56.8%–65.3%)	0

CCRT, concurrent chemoradiation therapy; ROI, region of interest; RT, radiation therapy; IC, induction chemotherapy; NAC, neoadjuvant chemotherapy.

*Law et al. did not report N-stage of patients and not included.

### Prognostic performance of DWI-MRI

**Figure 4:**
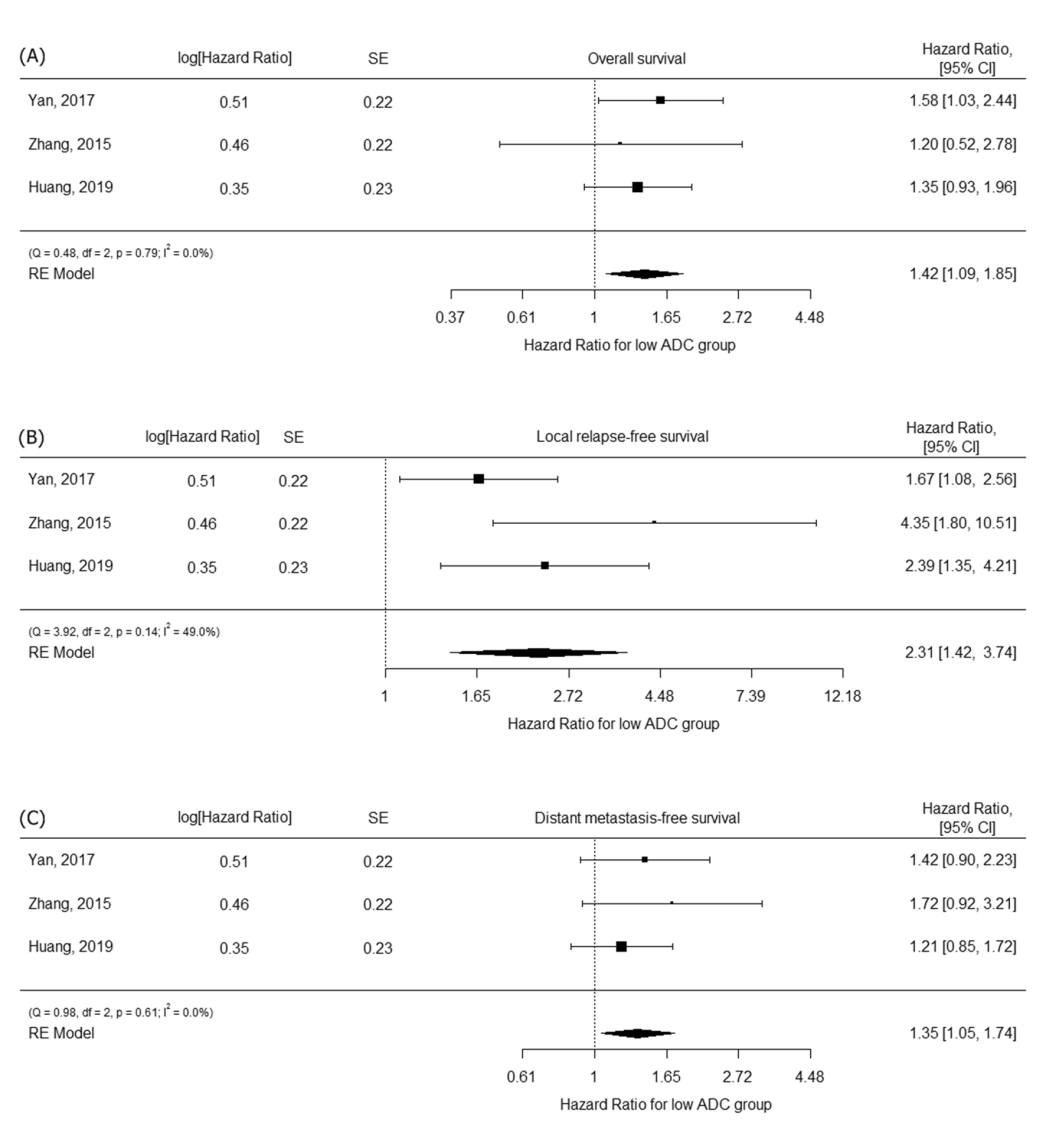
Forest plots for HRs of (**A**) overall survival, (**B**) local relapse-free survival, and (**C**) distant metastasis-free survival.

The HRs of OS, LRFS, and DMFS with respect to low ADC were evaluated in three studies^13,16,18^ (Fig. 4). The pooled HRs were 1.42 (95% CI 1.09–1.85) for OS, 2.31 (95% CI 1.42–3.74) for LRFS, and 1.35 (95% CI 1.05–1.74) for DMFS. Because no heterogeneity was present in OS and DMFS and only three studies were included, subgroup analysis was not performed.

## Discussion

This systematic review and meta-analysis assessed the predictive performance of pretreatment ADC for treatment response in patients with NPC. For the prediction of treatment response in NPC, pretreatment ADC showed pooled sensitivity of 87% and specificity of 70%. However, there was significant between-study heterogeneity regarding pooled sensitivity (*I*^2^ = 68.5%) and specificity (*I*^2^ = 92.3%). In the subgroup bivariate meta-regression analysis to investigate the source of heterogeneity, the studies that included a larger proportion of advanced T stage (> 70%), lower proportion of advanced N stage (≤ 70%), larger number of patients (> 60), used multiple b-values (≥ 4) for ADC mapping, and region of interest selection as either single section or volume had no heterogeneity (*I*^2^ = 0%). Additionally, low pretreatment ADC values were associated with worse OS, LRFS, and DMFS. Therefore, pretreatment ADC had a good predictive performance for treatment response, suggesting its potential role in guiding the treatment strategy in locoregionally advanced NPC.

In the subgroup analysis, no between-study heterogeneity was observed for studies consisting of patients with predominantly advanced T stages. Advanced T stage NPC usually manifests with skull base involvement or intracranial extension. Based on conventional MRI, the evaluation of skull base involvement in NPC has good diagnostic performance^4,36^; however, it has limitations for differentiating tumor involvement from reactive inflammation, leading to difficulties in accurately delineating the primary tumor^37^. In contrast, DWI reflects tissue water diffusivity^7^ with low pretreatment ADC values indicating biological features of tumor cells, such as hypoxia and higher cell density^16,38,39^. DWI performed better than conventional MRI for assessing the tumor microstructure^40^, making it useful for the evaluation of advanced T staged primary NPC. Moreover, because of the large amount of adipose tissue in the clivus, the diagnostic threshold of ADC in the skull base is higher than that of the nasopharynx^41^. Therefore, low ADC NPC values in the skull base (i.e., indicating advanced T stage) may provide a more consistent predictive performance than that in the nasopharynx.

In patients with advanced N stages, low pretreatment ADC values of primary tumors provide good predictive performance for detecting treatment response. The ADC value is reportedly directly inversely correlated with histological tumor grade^42–44^. High-grade tumors have higher cellular density with lower ADC values than low-grade tumors^45^. Moreover, patients with poorly differentiated or undifferentiated tumors (i.e. higher cellularity) are more likely to have metastatic cervical lymph nodes than patients with well-differentiated tumors^42^. Therefore, low ADC values in primary tumors may be associated with more frequent metastatic cervical lymph nodes making it a potential poor prognostic factor in NPC.

Traditionally, ADC is used to reflect tissue water diffusivity; however, it is no longer considered a true diffusion coefficient^7^. The concept of ADC is based on the Einstein equation which was calculated using the Gaussian law assuming water molecules move freely as in a glass of water^46^. However, cancer tissue interacts with cell membranes and surrounding macro- or micro-molecules making it non-Gaussian. To resolve this, intravoxel incoherent motion (IVIM) was introduced to reflect tissue perfusion and blood microcirculation in complex biological environments^46^. Consistent with our findings, tissue perfusion as measured by the bi-exponential fitting of multiple small b-values could presumably provide a more consistent result than the traditional mono-exponential fitting of ADC.

This study had some limitations. First, all except one study were published in China which might limit the generalizability of the results. Second, substantial heterogeneity was observed in the pooled specificity of treatment response prediction. However, the potential causes of between-study heterogeneity were explored via subgroup meta-regression. Third, we did not include studies investigating other prognostic factors, including clinical factors and molecular biomarkers. Recently, plasma Epstein-Barr virus DNA has also been proposed as a prognostic factor and screening tool in NPC^47,48^. Further studies using a combination of imaging techniques and molecular biomarkers for demonstrating treatment response in NPC may show more promising results for early treatment strategies. Fourth, for prognostic studies, subgroup analysis was not performed due to the small number of studies. However, the between-study heterogeneity was not substantial. Finally, the number of included studies was small which might have led to unstable results, particularly for the prognostic studies.

In conclusion, pretreatment ADC value was a good predictor of treatment response in NPC, providing clinically relevant information for developing early treatment strategies. The benefit of multiple b-values for the prediction of treatment response will need to be investigated further.

## Supplementary Information

Supplementary Figures.

## References

[1] Chen Y-P (2019). Nasopharyngeal carcinoma. Lancet.

[2] Yao J-J (2018). Comparing treatment outcomes of concurrent chemoradiotherapy with or without nimotuzumab in patients with locoregionally advanced nasopharyngeal carcinoma. Cancer Biol. Ther..

[3] Tang L-L (2017). Validation of the 8th edition of the UICC/AJCC staging system for nasopharyngeal carcinoma from endemic areas in the intensity-modulated radiotherapy era. J. Natl. Compr. Cancer Netw..

[4] Chen W-S (2016). Comparison of MRI, CT and 18F-FDG PET/CT in the diagnosis of local and metastatic of nasopharyngeal carcinomas: An updated meta analysis of clinical studies. Am. J. Transl. Res..

[5] Qin Y (2018). Predicting chemoradiotherapy response of nasopharyngeal carcinoma using texture features based on intravoxel incoherent motion diffusion-weighted imaging. Medicine (Baltimore).

[6] Hou J (2016). Value of intravoxel incoherent motion and dynamic contrast-enhanced MRI for predicting the early and short-term responses to chemoradiotherapy in nasopharyngeal carcinoma. Medicine (Baltimore).

[7] Iima M, Le Bihan D (2016). Clinical intravoxel incoherent motion and diffusion MR imaging: past, present, and future. Radiology.

[8] Hong J (2013). Value of magnetic resonance diffusion-weighted imaging for the prediction of radiosensitivity in nasopharyngeal carcinoma. Otolaryngol. Head Neck Surg..

[9] Zheng D (2013). Early assessment of induction chemotherapy response of nasopharyngeal carcinoma by pretreatment diffusion-weighted magnetic resonance imaging. J. Comput. Assist. Tomogr..

[10] Chen Y (2014). Diffusion-weighted magnetic resonance imaging for early response assessment of chemoradiotherapy in patients with nasopharyngeal carcinoma. Magn. Reson. Imaging.

[11] Hirshoren N (2019). Diffusion weighted magnetic resonance imaging of pre and post treatment nasopharyngeal carcinoma. Surg. Oncol..

[12] Tu N (2019). Treatment response prediction of nasopharyngeal carcinoma based on histogram analysis of diffusional kurtosis imaging. AJNR Am J Neuroradiol.

[13] Yan DF (2017). The prognostic value of pretreatment tumor apparent diffusion coefficient values in nasopharyngeal carcinoma. BMC Cancer.

[14] Liu J (2016). Use of texture analysis based on contrast-enhanced MRI to predict treatment response to chemoradiotherapy in nasopharyngeal carcinoma. J. Magn. Reson. Imaging.

[15] Xiao-ping Y (2016). Intravoxel incoherent motion MRI for predicting early response to induction chemotherapy and chemoradiotherapy in patients with nasopharyngeal carcinoma. J. Magn. Reson. Imaging.

[16] Zhang Y (2015). Prognostic value of the primary lesion apparent diffusion coefficient (ADC) in nasopharyngeal carcinoma: A retrospective study of 541 cases. Sci. Rep..

[17] Chen Y (2015). Diffusion kurtosis imaging predicts neoadjuvant chemotherapy responses within 4 days in advanced nasopharyngeal carcinoma patients. J. Magn. Reson. Imaging.

[18] Huang TX (2019). The primary lesion apparent diffusion coefficient is a prognostic factor for locoregionally advanced nasopharyngeal carcinoma: A retrospective study. BMC Cancer.

[19] Law BKH (2016). Diffusion-weighted imaging of nasopharyngeal carcinoma: Can pretreatment DWI predict local failure based on long-term outcome?. Am. J. Neuroradiol..

[20] Moher D, Liberati A, Tetzlaff J, Altman DG (2009). Preferred reporting items for systematic reviews and meta-analyses: The PRISMA statement. Ann. Intern. Med..

[21] Oldenhuis C, Oosting S, Gietema J, De Vries E (2008). Prognostic versus predictive value of biomarkers in oncology. Eur. J. Cancer.

[22] Whiting PF (2011). QUADAS-2: A revised tool for the quality assessment of diagnostic accuracy studies. Ann. Intern. Med..

[23] Fan WJ (2020). Diffusion-weighted imaging as a follow-up modality for evaluation of major salivary gland function in nasopharyngeal carcinoma patients: A preliminary study. Strahlenther Onkol..

[24] Deeks JJ, Macaskill P, Irwig L (2005). The performance of tests of publication bias and other sample size effects in systematic reviews of diagnostic test accuracy was assessed. J. Clin. Epidemiol..

[25] Hoaglin DC (2016). Misunderstandings about Q and ‘Cochran's Q test'in meta-analysis. Stat. Med..

[26] Kim KW, Lee J, Choi SH, Huh J, Park SH (2015). Systematic review and meta-analysis of studies evaluating diagnostic test accuracy: A practical review for clinical researchers—Part I. General guidance and tips. Korean J. Radiol..

[27] Devillé WL (2002). Conducting systematic reviews of diagnostic studies: Didactic guidelines. BMC Med. Res. Methodol..

[28] Huang W (2019). Potential value of non-echo-planar diffusion-weighted imaging of the nasopharynx: A primary study for differential diagnosis between recurrent nasopharyngeal carcinoma and post-chemoradiation fibrosis. Acta Radiol..

[29] Zhang GY (2015). Pretreatment diffusion-weighted MRI can predict the response to neoadjuvant chemotherapy in patients with nasopharyngeal carcinoma. Biomed. Res. Int..

[30] Huang WY (2018). In vivo imaging markers for prediction of radiotherapy response in patients with nasopharyngeal carcinoma: RESOLVE DWI versus DKI. Sci. Rep..

[31] Xiao Y (2019). Longitudinal assessment of intravoxel incoherent motion diffusion weighted imaging in evaluating the radio-sensitivity of nasopharyngeal carcinoma treated with intensity-modulated radiation therapy. Cancer Res. Treat..

[32] Xiao Y (2015). Intravoxel incoherent motion-magnetic resonance imaging as an early predictor of treatment response to neoadjuvant chemotherapy in locoregionally advanced nasopharyngeal carcinoma. Medicine (Baltimore).

[33] Qamar S (2020). Pre-treatment intravoxel incoherent motion diffusion-weighted imaging predicts treatment outcome in nasopharyngeal carcinoma. Eur. J. Radiol..

[34] Lu L, Li Y, Li W (2016). The role of intravoxel incoherent motion MRI in predicting early treatment response to chemoradiation for metastatic lymph nodes in nasopharyngeal carcinoma. Adv. Ther..

[35] Hu Y (2016). Predictive value of diffusion-weighted magnetic resonance imaging for cervical lymph node metastasis in nasopharyngeal carcinoma after chemoradiotherapy. Int. J. Clin. Exp. Med..

[36] Chung NN, Ting LL, Hsu WC, Lui LT, Wang PM (2004). Impact of magnetic resonance imaging versus CT on nasopharyngeal carcinoma: Primary tumor target delineation for radiotherapy. Head Neck.

[37] Altun M, Tenekeci N, Kaytan E, Meral R (2000). Locally advanced nasopharyngeal carcinoma: computed tomography findings, clinical evaluation, and treatment outcome*. Int. J. Radiat. Oncol.* Biol.* Phys..

[38] Ni X (2015). Diffusion-weighted magnetic resonance imaging in predicting the radiosensitivity of cervical cancer. Int. J. Clin. Exp. Med..

[39] Larocque MP, Syme A, Allalunis-Turner J, Fallone BG (2010). ADC response to radiation therapy correlates with induced changes in radiosensitivity. Med. Phys..

[40] Zheng X (2020). Diffusion kurtosis imaging and tumour microstructure for monitoring response to radiotherapy in human nasopharyngeal carcinoma xenografts. Jpn. J. Clin. Oncol..

[41] Ginat DT, Mangla R, Yeaney G, Johnson M, Ekholm S (2012). Diffusion-weighted imaging for differentiating benign from malignant skull lesions and correlation with cell density. Am. J. Roentgenol..

[42] Razek AAKA, Kamal E (2013). Nasopharyngeal carcinoma: correlation of apparent diffusion coefficient value with prognostic parameters. Radiol. Med. (Torino).

[43] Ichikawa Y, Sumi M, Sasaki M, Sumi T, Nakamura T (2012). Efficacy of diffusion-weighted imaging for the differentiation between lymphomas and carcinomas of the nasopharynx and oropharynx: Correlations of apparent diffusion coefficients and histologic features. Am. J. Neuroradiol..

[44] Razek AA, Elkhamary S, Al-Mesfer S, Alkatan H (2012). Correlation of apparent diffusion coefficient at 3T with prognostic parameters of retinoblastoma. Am. J. Neuroradiol..

[45] Razek AAKA (2010). Diffusion-weighted magnetic resonance imaging of head and neck. J. Comput. Assist. Tomogr..

[46] Le Bihan D (1988). Separation of diffusion and perfusion in intravoxel incoherent motion MR imaging. Radiology.

[47] Chan KA (2017). Analysis of plasma Epstein-Barr virus DNA to screen for nasopharyngeal cancer. N. Engl. J. Med..

[48] Tang L-Q (2016). Establishment and validation of prognostic nomograms for endemic nasopharyngeal carcinoma. JNCI J. Natl. Cancer Inst..

