# Supplementary material for: Diffusion-weighted MRI for predicting treatment response in patients with nasopharyngeal carcinoma: a systematic review and meta-analysis
Source: Sci Rep. 2021 Sep 23;11:18986. doi: 10.1038/s41598-021-98508-5 (PMC8460673; doi:10.1038/s41598-021-98508-5)

**Supplemental Materials for**

**Diffusion-weighted MRI for predicting treatment response and prognosis in patients with nasopharyngeal carcinoma: a systematic review and meta-analysis**

Min Kyung Lee, MD^1^, Yangsean Choi, MD^2*^, So-Lyung Jung, MD, PhD^1^

^1^Department of Radiology, Yeouido St. Mary’s Hospital, College of Medicine, The Catholic University of Korea, Seoul, Republic of Korea

^2^Department of Radiology, Seoul St. Mary’s Hospital, College of Medicine, The Catholic University of Korea, Seoul, Republic of Korea

**Corresponding author:** Yangsean Choi, M.D.

Department of Radiology, Department of Radiology, Seoul St. Mary’s Hospital

College of Medicine, The Catholic University of Korea

Seoul, Republic of Korea

Phone: 82-2-2258-1442

E-mail address: phillipchoi007@gmail.com

**Supplemental Figure 1.** Quality assessment using (a) the Quality Assessment of Diagnostic Accuracy Studies-2 (QUADAS-2) and (b) Quality in Prognosis Studies (QUIPS).


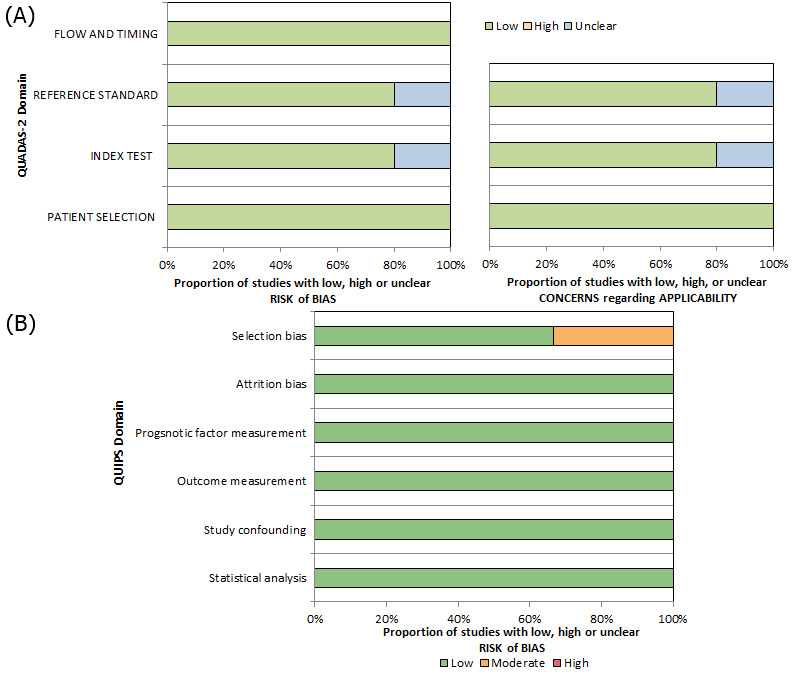


**Supplemental Figure 2.** Deek’s funnel plot


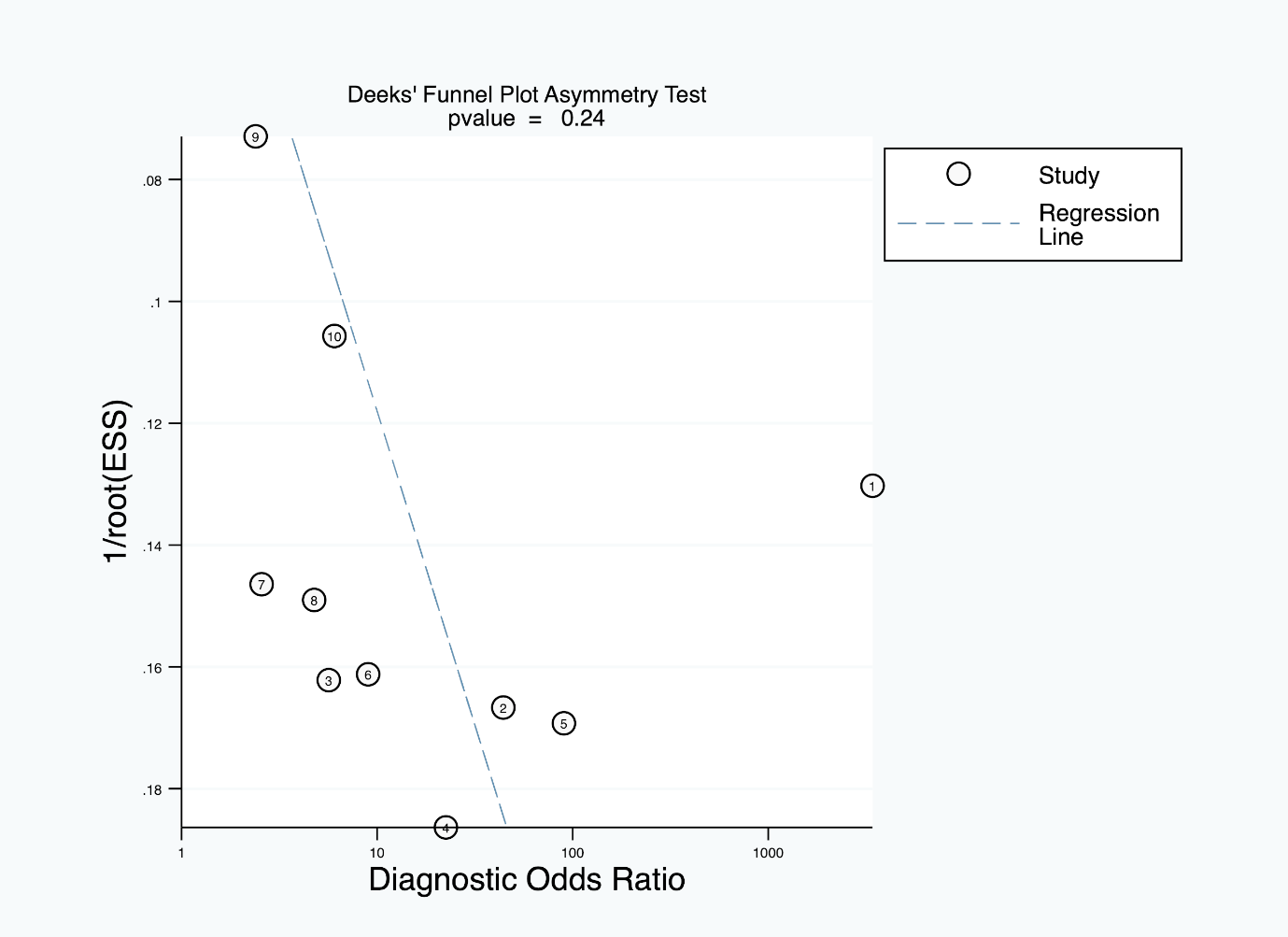

Supplement: Supplementary file 1 — Supplementary Figures. [file 41598_2021_98508_MOESM1_ESM.docx]
